# Towards Autonomous Modular UAV Missions: The Detection, Geo-Location and Landing Paradigm

**DOI:** 10.3390/s16111844

**Published:** 2016-11-03

**Authors:** Sarantis Kyristsis, Angelos Antonopoulos, Theofilos Chanialakis, Emmanouel Stefanakis, Christos Linardos, Achilles Tripolitsiotis, Panagiotis Partsinevelos

**Affiliations:** 1 School of Mineral Resources Engineering, Technical University of Crete, Chania 73100, Greece; sarkyritsis@gmail.com (S.K.); linardos_thegreat@hotmail.com (C.L.); atripol@mred.tuc.gr (A.T.); 2 School of Electrical and Computer Engineering, Technical University of Crete, Chania 73100, Greece; a_antonopoulos@outlook.com (A.A.); theofilos13@hotmail.com (T.C.); emmanuelstef95@gmail.com (E.S.); 3 Space Geomatica Ltd., Chania 73133, Greece; 4 School of Mineral Resources Engineering, Laboratory of Geodesy and Geomatics Engineering, SenseLAB Research Group, Chania 73100, Greece

**Keywords:** UAV, search and rescue, autonomous landing, smart-phone drone

## Abstract

Nowadays, various unmanned aerial vehicle (UAV) applications become increasingly demanding since they require real-time, autonomous and intelligent functions. Towards this end, in the present study, a fully autonomous UAV scenario is implemented, including the tasks of area scanning, target recognition, geo-location, monitoring, following and finally landing on a high speed moving platform. The underlying methodology includes AprilTag target identification through Graphics Processing Unit (GPU) parallelized processing, image processing and several optimized locations and approach algorithms employing gimbal movement, Global Navigation Satellite System (GNSS) readings and UAV navigation. For the experimentation, a commercial and a custom made quad-copter prototype were used, portraying a high and a low-computational embedded platform alternative. Among the successful targeting and follow procedures, it is shown that the landing approach can be successfully performed even under high platform speeds.

## 1. Introduction

The global commercial unmanned aerial vehicle (UAV) market has shown considerable growth [1], gradually embracing a wide range of applications. Irrespective of the underlying application (media and entertainment, energy, government, agriculture, search and rescue (SAR), environment, etc.), the majority of common UAV operations involve image and video acquisition, maneuvering, navigational attitude, routing to predefined waypoints, landing and failsafe features, which are processed and fulfilled through the standard flight controller systems. Nevertheless, in order to address a broader range of applications, one has to migrate to integrated processing add-ons that would carry out on demand, on-board, collaborative or autonomous functions, towards an intelligent UAV functionality. Increasing the computational potential of the UAV does not solely address computationally demanding tasks, but transforms the UAV into a potential decision operating system that can take control of its own flight and perform optimized missions.

The significance of UAV autonomy and processing power is demonstrated in several applications including technical infrastructure inspection [2,3,4,5,6], indoor navigation using simultaneous localization and mapping [7,8,9,10], obstacle avoidance [11,12,13,14], terrain reconstruction [15,16,17], and real-time environmental monitoring [18,19]. The flexibility, human safety, cost effectiveness and ease of use facilitates the usage of UAVs to support humanitarian actions in disaster situations [20,21]. The identification and rescue of potential victims in the shortest possible time constitutes the main objective of search and rescue operations [22,23].

In the current study, we address an autonomous operating scenario of a UAV that takes off, performs an area scan, and finally lands onto a moving platform. This concept was partially addressed in an ongoing challenge by DJI (2016 DJI Developer Challenge). More specifically, the overall scenario includes a UAV that takes off from a moving platform to extract, recognize, track and follow possible targets where markers in the form of AprilTags are scattered to represent survivors (true IDs) and non-survivors (false IDs). After real-time geo-location of the survivors through geographic coordinates, the UAV approaches, follows and autonomously lands on the moving platform. Both the UAV and platform are equipped with a Global Navigation Satellite System (GNSS). Landing on a high-speed moving platform constitutes a highly demanding task and is addressed in several studies under varying environments. In [24], optical flow is used for indoor landing of a vertical takeoff and landing (VTOL) UAV on a moving platform. Apparently, the platform was moving manually at low speeds only in the vertical direction. No details are given about the respective speed, but this optical flow approach was simulated with a speed most certainly under 6 m/s. An algorithm based on robust Kalman filters for vision-based landing of a helicopter on a moving target is given in [25]. Extended Kalman and Extended H_∞_ filters were used in [26] to fuse visual measurements with inertial data in order to maintain a high sampling rate and cover short-period occlusions of the target. This algorithm assumes that when in hover, the aerial vehicle presents zero roll, pitch and yaw values, along with zero movement in the northing and easting. However, as the authors also recognize, this is almost impossible to achieve in real life. IR light pattern detection is used in [27], but the landing platform was moving with a speed as low as 40 cm/s. In [28], a rope is used to link the UAV with a moving platform and sensor fusion is applied to provide the relative position and velocity state estimation. A similar tethering approach is presented in [29]. More recently, linear programming-based path planning is proposed in [30], whereas the usage of an omnidirectional camera for outdoor autonomous landing on a moving platform is investigated in [31].

The velocity and size of the moving platform are determinative parameters on the success of the previous approaches, especially when different types (i.e., car, ship, etc.) of platforms are examined. The size of a UAV landing onboard a ship (i.e., [32,33,34,35]) is several levels of magnitude smaller than the available landing space. At the same time, to the best of our knowledge, there are only a few studies that propose techniques for automated UAV landing on fast moving (i.e., >20 km/h) cars. For example, in [36], an unmanned aircraft travelling at 75 km/h lands on the roof of a moving car with the assistance of a relatively large landing pad that seems impractical to be attached in common cars, while the car speed provides a moving air runway that actually facilitates landing for these types of UAVs.

The present work differs from previous studies since the vehicle size is comparable to the landing platform dimensions and the landing platform moves at more than 20 km/h. Furthermore, the main contributions of this work include: (1) improvement of visual marker detection rate to 30 frames per second; (2) accurate target geolocation; (3) implementation of a novel “aggressive” landing approach technique inspired from standard practices of airplane pilots; and (4) implementation of a low-computational landing approach with our “smart-drone” prototype setting, which constitutes a real revolution towards the delivery of a low-cost, small size personalized UAV with enhanced capabilities, not even tackled from large scale commercial UAVs.

The rest of the paper is structured as follows: Section 2 presents the hardware and software components used in the underlying approaches, followed by Section 3 where the identification and geo-location methodology is detailed. In Section 4, the implementation of the follow-up and landing on a high speed moving platform is presented along with the processing capabilities of a UAV smart-phone device system to provide a low-cost, modular alternative to commercial UAV products. Finally, in Section 5, the main findings of the present study and future plans are discussed.

## 2. Materials and Methods

**Figure 1:**
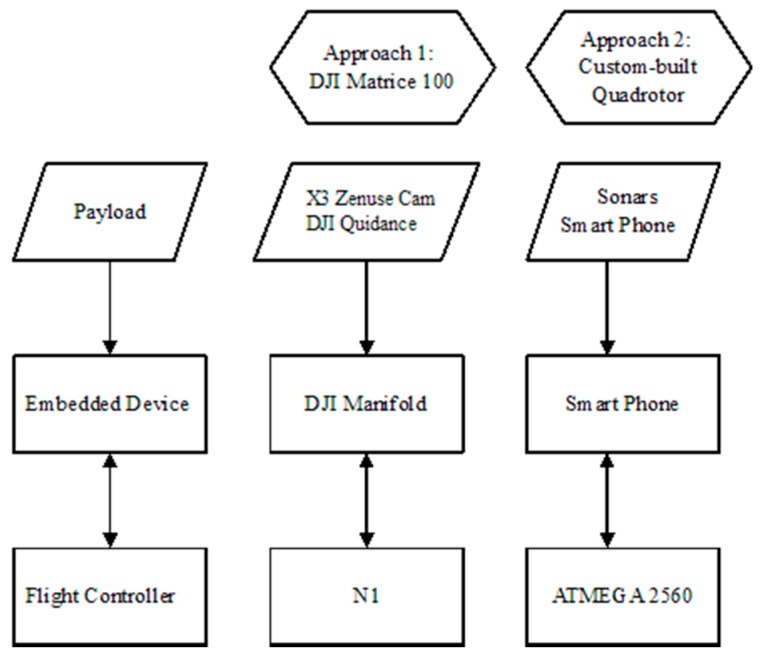
The general architectural design for the two alternative hardware/software approaches.

In the subsequently described approaches, we use two different hardware and software settings representing a high and low computational potential of the underlying processing power. Both scenarios are based on the same architectural design (Figure 1). The flight controller (FC) of the UAV is responsible for the flying attitude and stability of the vehicle. An embedded device interacts with the FC and the peripheral devices (i.e., camera, sonars). The embedded device collects the data from the peripherals, and after processing, it decides about the movement of the UAV (i.e., obstacle avoidance, landing pad detection). The decisions are forwarded to the FC as flight commands (i.e., moving to x, y, z or starting to descend at x m/s).

In the first scenario, the following hardware and software components were used: a DJI Matrice 100 UAV platform (Shenzhen, China), X3 Zenmuse camera (Shenzhen, China) with a gimbal and 4 k @ 30 fps and 1080 p @ 60 fps, a NVIDIA Tegra K1 SOC embedded processor (Santa Clara, CA, USA) (DJI Manifold component), and the DJI Guidance Sensor (Shenzhen, China). In the second scenario, a Quadrotor UAV (Senselab, Chania, Greece), an ATMEGA 2560 flight controller (San Jose, CA, USA) and the processing power of a mobile device are used. The latter constitutes our “smart-drone” implementation [37], which integrates a standard smart-phone into a custom made UAV carrier. The DJI Matrice 100 comes with the A2 GPS PRO Plus GPS receiver (Shenzhen, China), whereas the custom built “smart-drone” setting is powered by the smart-phone’s GPS receiver. Apparently, custom settings may incorporate more advanced multi-frequency, multi-constellation GNSS boards if sub-centimeter accuracy in AprilTag geolocation is required, yet this implementation is far beyond the scope of the current study.

**Figure 2:**
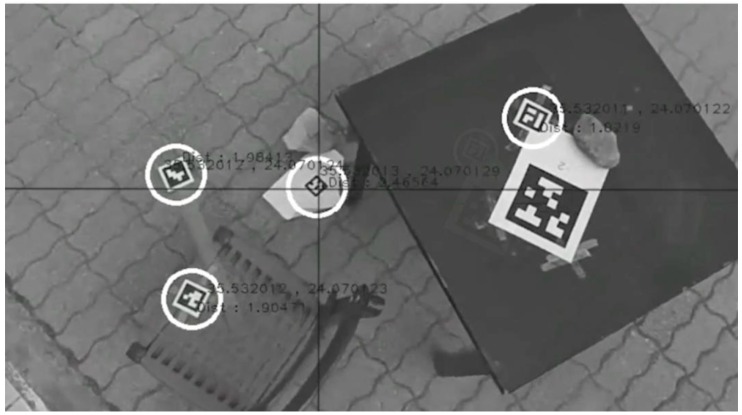
Multiple AprilTag detection, identification and geo-location. The large AprilTag on the right is not detected as its identity is not included in the survivor IDs.

Various types and sizes of AprilTags are used to denote the survivors and landing targets. The AprilTag markers [38] are high-contrast, two-dimensional tags designed to be robust to low image resolution, occlusions, rotations, and lighting variation [38] that have been used in several UAV-related applications [9,39,40] (Figure 2). Other 2D markers such as ARTag [41] have been used for UAV localization [42]; however, in this work, we used AprilTag mainly due to the restrictions set by the aforementioned challenge. However, our approach can be easily adapted to work with other tags.

In both scenarios, the 25h9 AprilTags with dimensions of 6 cm × 6 cm are used to identify the victim, whereas the 16h9 and 36h11 tags with dimensions 40 cm × 40 cm are used to determine the landing platform. In the first scenario, the camera operates in 4 k @ 30 fps and 1080 p @ 60 fps, although in our application, the feed that was retrieved was 720 p @ 60 fps. In the second scenario, the majority of the smart-phone cameras record 1080 p @ 30 fps videos, although several devices support 1080 p @ 60 fps.

The software was developed within the Robotic Operating System (ROS) environment using OpenCV functions. In the first scenario, the DJI libraries for the connection with the drone and camera were utilized, while in the second scenario, the application was implemented on the Android OS.

As described above, the current implementation refers mainly to multi-copters, the majority of which navigate under a tilted fashion, making landing while moving, a non-trivial task. Furthermore, the landing area is considered comparable to the UAV size (i.e., car roof-top), making experimentation and testing quite challenging.

**Figure 3:**
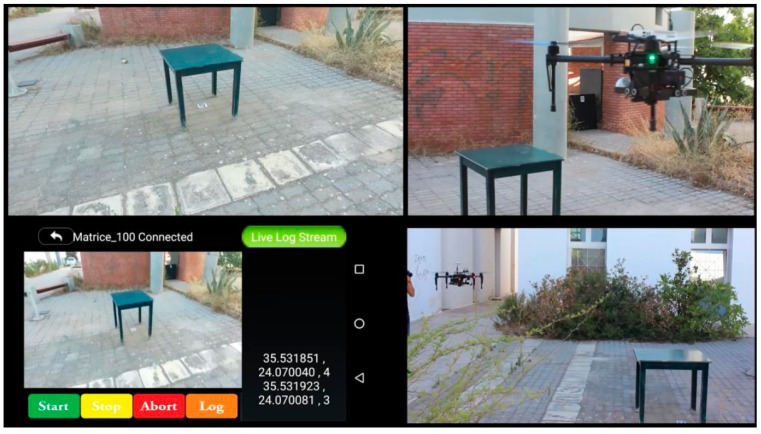
Scanning and sequential AprilTag detection, identification and geo-location. The mobile application screenshot shows survivor coordinates and IDs.

In order for a user to be able to use the described capabilities, an Android application is implemented (Figure 3, down-left). Through this application, the end user can mark a survey area on a map. The UAV takes off from the moving platform, scans the full area, identifies the required targets; while upon completion, it seeks the landing platform. Using the camera feed, it tries to detect the landing target while approaching the GPS coordinates of the platform. Once the platform has been detected, the drone follows and starts descending towards the platform’s landing pad.

## 3. Search, Detection and Geo-Location Implementation and Results

### 3.1. Target Recognition of Survivor AprilTags

As described by the implementation scenario, the UAV, after taking off from the moving platform, navigates towards the search area, where markers in the form of AprilTags are scattered to represent victims (true IDs) and non-victims (false IDs). According to the AprilTag size and placement, flying height, gimbal speed and camera mode (resolution vs. frame rate), the UAV adjusts its speed to accomplish a safe frame acquisition. Each acquired frame by the camera is forwarded to the embedded processor, where the dedicated ROS node processes it in order to detect the AprilTag.

**Figure 4:**
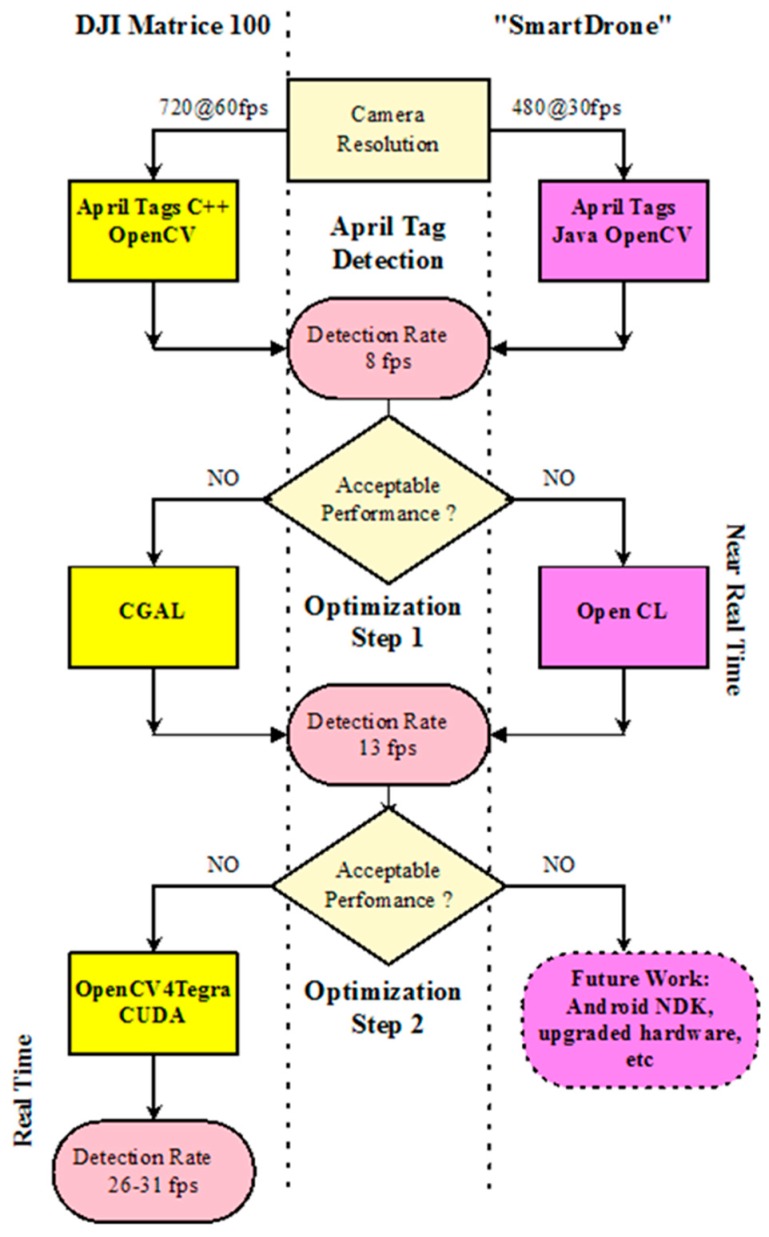
AprilTag detection flowchart for the two settings (DJI Matrice 100 and custom “smart-drone”). With the first setting and after two optimization steps, a detection rate of 26–31 fps was accomplished, whereas the second solution accomplishes a detection rate of 13 fps.

We focused our efforts on implementing a high frame rate detection approach (i.e., ~30 fps), enabling the survivor detection in real-time. Thus, several computational approaches were undertaken (Figure 4):

Initially, the AprilTags C++ library [43] that is available in open source under GNU LPGL v2.1 [44] was employed. This detection approach was successful in identifying and locating the AprilTags but within a low frame rate, mainly due to its poor computational performance.

The second detection approach included the OpenCV implementation based on the solution provided in [44]. Under this implementation, AprilTag detection and recognition was accomplished under a rate of about eight frames per second.

Although the frame rate was greatly improved, in order to further improve the performance of the algorithm, we used the CGal-based approach available in [45] to detect quads. This approach gave a great performance upgrade, resulting in 13 frames per second, and the results seemed to be near real-time.

To further enhance the system’s performance, we parallelized the process as Graphics Processing Unit (GPU) functions. This seems to be viable, as the detection process described in [38] does not involve sophisticated algorithms that demand large local memory [46]. A similar approach was proposed for QR tags in [47]. Therefore, we used the OpenCV4Tegra [48] framework that permits performance of all OpenCV functions in parallel as GPU functions. However, some of the functions described in [38] are computationally expensive, and, furthermore, they are not available in OpenCV. For example, the “Find Union” function iteratively constructed disjoint subsets inside the initial pixel set of the image, resulting in a processing bottleneck. To overcome such computationally demanding tasks, the CUDA parallel computing platform [49] was employed along with appropriate modifications to the implementation.

**Figure 5:**
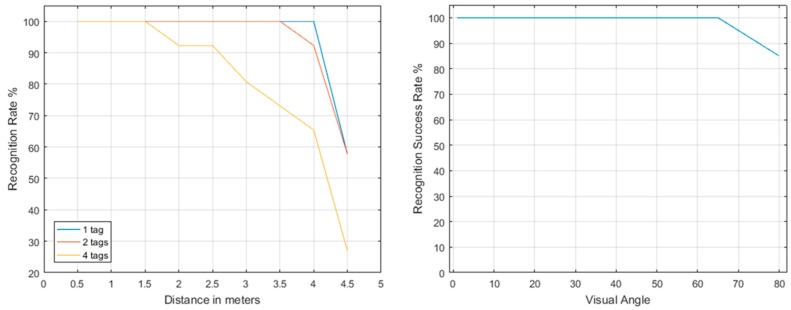
**Left:** the detection success rate for multiple (1, 2 and 4 AprilTags) targets and distances (UAV to target); **Right:** the detection success rate for single target per visual angle.

The final AprilTag detection optimization was performed at a rate of 26–31 frames per second. In addition, 6 cm × 6 cm moving AprilTags were detected and identified at a distance of about 4 m, even at extreme visual angles (Figure 5). Single false positive detections occur rarely, while more than one “true positive” frame recognitions verify the AprilTag ID, making the overall false positive occasions obsolete. When the wind, UAV or survivor movements are considerable, some disruptions in the detection occur, yet they do not contaminate the real-time processing of the implementation. In any case, approaches such as the one presented in [50] for the control of the UAV velocity and attitude under various disturbances may be further incorporated into the overall system architecture.

### 3.2. Geo-Location of Survivor AprilTags

The coordinates of the identified AprilTags are determined via the image geometry, gimbal orientation and the UAV GPS readings. However, as an object moves away from the principal point of the frame, large distortions occur in the image geometry. Thus, after the initial identification of the AprilTag, the gimbal moves to position it in the center of the camera’s field-of-view (FOV), the principal point. The detection algorithm uses homography pose estimation to extract the translation and rotation of the AprilTag’s center coordinates with respect to the image center, AprilTag size, and the camera’s intrinsic and extrinsic characteristics. The latter are derived through dedicated camera calibration, performed either offline or even online using: (a) the DJI API (Application Programming Intreface) calls for acquiring camera data; and (b) well known implementations using OpenCV. The AprilTag coordinates are generally calculated relative to the camera center.

In order to provide geographical coordinates to the AprilTag, we have to take into account that three different coordinate systems exist: (1) the 3D UAV system; (2) the gimbal mounted under the frame that houses the camera; and (3) the 2D image coordinates. Thus, the problem is obtaining the world coordinates of the AprilTag marker given its image coordinates. Thus, the following approach was implemented to transform the camera’s to the UAV’s reference system: when a new frame is available for processing, the gimbal and compass values are recorded and their angular distance is calculated. Therefore, the target’s distance and orientation with respect to the UAV can be computed, knowing the geometry of the UAV and its components. As a final step, the UAV’s GPS records are used as inputs into the Vincenty’s Direct Formulae [51] along with the target’s distance and orientation provided in the previous step. These formulae deliver the target’s GPS coordinates.

Furthermore, in the case of multiple AprilTag detection, the identification and localization initiates by sequentially detecting the closest to the principal point AprilTag, centering it through the camera-gimbal movement and providing its coordinates in real-time (Figure 2). Then, the specific AprilTag ID is excluded from further searching, and the next one is consecutively centered providing its coordinates. Thus, step by step, all AprilTags are identified only once.

### 3.3. Area Scanning Approach

The navigation approach in terms of the routing and scanning schedule is a key task that needs to be optimized. A first approach divides the search area in parallel adjacent flying strips, a well-known geometry-based photogrammetric task. Based on the camera’s focal length, flying height, camera’s pixel resolution (x and y), side-lap, angular FOV, target scaling factor, etc., the scan lines can be easily estimated.

The UAV initially retains a constant flight height, determined by the size of the AprilTags with the use of the barometer and sonars onboard. Occlusion and obstacles are undertaken through an obstacle avoidance system (e.g., MB1230 sonar sensor, MaxBotix, Brainerd, MN, USA,) or by determining a height map—elevation model of the area through a sensor system (e.g., DJI Guidance). According to the obstacle avoidance readings, the UAV adjusts its altitude not only to avoid the obstacles but also to lower its elevation and acquire a better view of occluded areas. Under the lowered or elevated positions, a full camera rotation is performed in order to include as many occluded AprilTags as possible for an optimized visibility scan (Figure 3). The rotation is facilitated by using both gimbal and drone attitude.

## 4. Takeoff and Landing Approach

**Figure 6:**
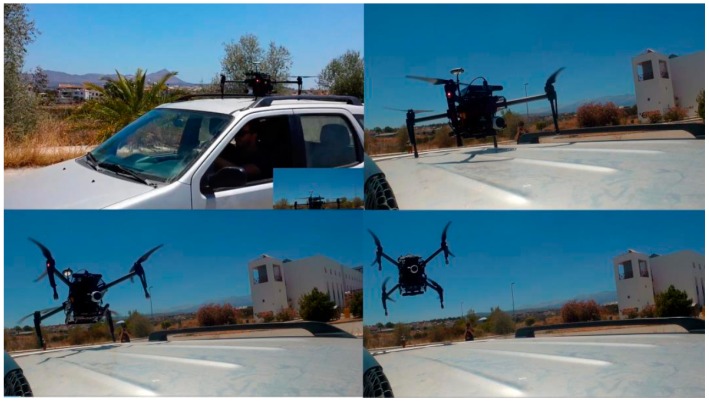
UAV taking off of a moving vehicle.

Independent of the UAV setting used (i.e., DJI Matrice 100 or “smart-drone”) takeoff is a straightforward task and no problems were identified since the flight controller, in both settings, is able to manage the stabilization issues that occur when taking off from a moving platform, tested up to 30 km/h (Figure 6). However, distinct procedures for landing have been taken into consideration.

### 4.1. High-Computational Processing Setting

In this case, when the landing target is stationary, the actual landing point is within a few centimeters (1–5 cm) from the expected one. When the landing is performed on a moving platform, three distinct approaches were implemented, one based on *follow*, one on *aggressive*, and a third on an integrated *hybrid* mode.

**Figure 7:**
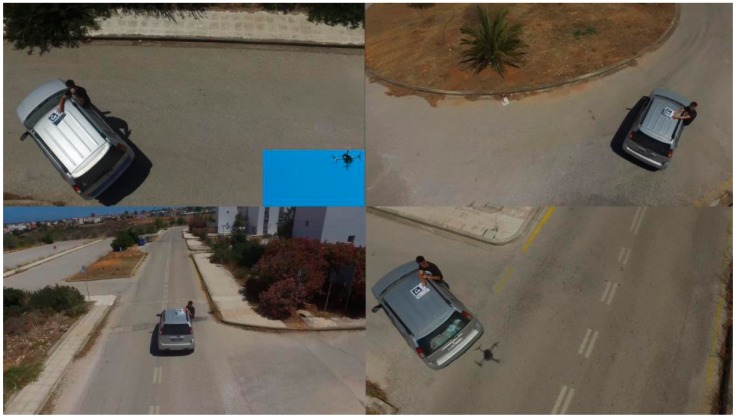
The *following* approach under extreme turning and speed sequences.

According to the *follow* mode, the UAV follows the moving platform from a safe distance and flying height according to the GPS readings of the platform, until the camera locks the AprilTag landing pattern (Figure 7). The approach of the UAV is gradual, meaning that it lowers its altitude, while the AprilTag remains targeted until it reaches the landing pad. If, for any reason, the AprilTag is lost for a predefined frame number, the UAV rises to retarget the AprilTag.

Under the *aggressive* approach, the UAV hovers over the area of interest and approximates the platform’s relative angular position moving trajectory through the GPS and then uses the camera to target and lock in the moving platform. This approach uses real-time error correction and trajectory planning, achieved with the use of the OpenCV, ROS API, and DJI API calls. In this approach, the drone detects the vehicle, directly targets the theoretical predicted intersection between their courses, and follows the closest distance approach. The UAV then navigates in a direct approach towards the platform.

The *hybrid* approach takes advantage of the speed and optimization of the *aggressive* approach, until the platform is close enough to identify the landing AprilTag. Then, the landing procedure of the *follow* approach is performed. Thus, the UAV selects the shortest path to the target, and at the final stage of approach, tries to hover above the target and slowly descend into landing.

**Figure 8:**
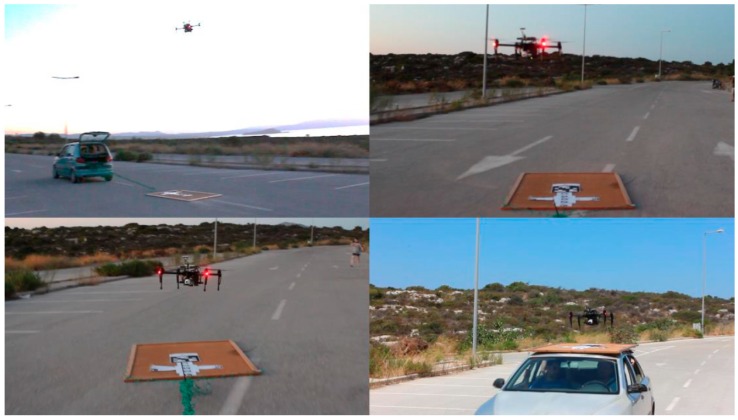
UAV landing on a moving platform.

All approaches were tested under various platform speeds and conditions. We had successful touchdowns from stationary targets to moving targets, with speeds reaching 30 km/h. The moving target scenarios tested included both following a target and landing, and also locating a distant target, reaching it and then landing (Figure 8). In our testing, all landings were successful in approaching the platform; however, at high speeds and wide platform turning angles, the final 25 cm of descent resulted sometimes in less accurate landings and instability.

Therefore, depending on the moving platform type, when its speed reaches more than 20 km/h, a stabilizing practice should be devised in order to alleviate the gradual friction of the UAV landing legs that may lead to unstable landing or even crashing. Such implementations may include reconfigurable perching elements [52], electromagnets in landing legs or platform, fastening straps, retractable hooks, etc.

### 4.2. Low-Computational Processing Setting 

The previous section detailed the procedures for landing a UAV powered with a high-computational processing unit on a moving platform. In this section, we discuss the case of the smart-drone unit, where the obstacle avoidance and landing procedures need to be revisited.

**Figure 9:**
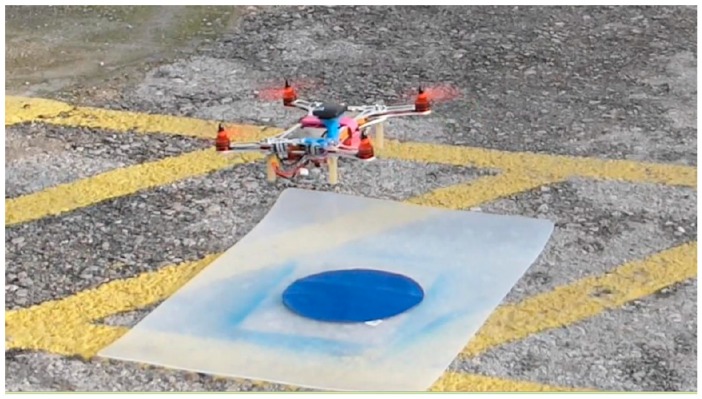
Smart-drone prototype and corresponding landing pad.

In order to detect the landing base, real-time image processing takes place in the smart-drone’s mobile device (Figure 9). More specifically, the acquired feed from the smart-phone camera is processed in real-time to detect the landing base. The base is designed as a discrete circle on a homogenous background that is colored in such a way that it can be easily distinguished from the surroundings. From an algorithmic point of view, each acquired still frame from the image stream is transformed to HSV (Hue, Saturation, Value), and the contours that are relative to the target’s specific HSV values are calculated. After this filtering, the circle areas in the image are determined through the Hough circles function, so that that the base can be identified. In order to estimate the UAV distance from the landing base, the pose of the target circle needs to be determined. When facing the target at a 90° angle, the distance is estimated by calculating the target size in pixels and referencing it to its calibrated values. When facing from a different angle, we calculate the homography and extract the rotation and translation of the target. Thus, while the exact position of the object into the 3D space and its projection to a 2D surface are determined, we estimate the distance by considering the pixel area of the target.

The processing is undertaken by the smart-phone processor, and flight correction data are forwarded to the flight controller in order to change its position to new relative (x, y, z) coordinates and descend at a given rate, until the UAV reaches the landing pad.

## 5. Discussion and Conclusions

In this study, we presented a UAV related scenario representing an abundance of applications characterized by search, detect, geo-locate and automatic landing components. The key function of our approach is the UAV computational autonomy, which adjusts its attitude according to the specific environment. The underlying application adheres to a Search and Rescue paradigm, where the UAV takes off, scans a predefined area, geo-locates possible survivor targets, and, upon completion, returns to land on a moving platform. In order to support computational autonomy and perform “expensive” real-time tasks, we integrated high and relatively low-computational embedded systems.

Both implementations were able to identify the targets and proceeded successfully to the landing platforms. The first approach, however, proved more reliable, as it was able to keep on tracking and approaching in real-time, fast moving landing targets. The low-computational smart-drone approach seems very promising, but still needs improvement in the stabilization system of the phone’s camera. Specifically, a gimbal-like mechanism needs to be constructed specifically for the mobile phone.

The challenging tasks of the described scenario include the real-time target detection and identification, and the follow, approach and landing implementations in the moving platform. Real time target detection was accomplished using a GPU parallelization, in order to achieve 30 fps processing speed. The significance of the processing frame rate is justified due to the high speed landing platforms, under which our implementation succeeded with following and landing upon. In several other studies, either the platform velocity was low, or the size of the moving platform was considerably large in order to enable a safe landing [32,33,34,35]. To the best of our knowledge, there are only a few studies that propose techniques for automated UAV landing on fast moving (i.e., >20 km/h) cars [35]. Even then, the platform speed facilitates landing of airplane-like UAVs, since it actually provides a moving air runway.

For multi-copters and relatively small landing areas, our implementation provides a novel approach scheme. Thus, our “aggressive” approach for the multi-copters is used to provide a robust and direct way for the UAV to reach the landing target. Furthermore, the usual “follow” mode used in recreation or most of the commercial follow-me approaches remains dangerous when occluded, and vegetated areas are evident since the UAV will adjust its attitude relative to the moving target under a lagged approach. The “aggressive” approach, when utilized after the landing target camera locking, ensures an obstacle-free corridor towards a safe landing.

Another subtle detail of our approach is the real-time geo-location capability that provides geographic coordinates under an optimized implementation. Since camera distortion may provide inaccurate location estimations, principal point targeting, pose extraction and calibration are used to provide more accurate coordinates. Geo-location is crucial especially in SAR applications in large regions, such as the ocean, where multiple people may be in danger. The exact location of multiple people is not achieved by today’s means, since the common practice involves merely visual inspection, especially when UAVs fly relatively high. Under our approach, the authorities along with the rescue and recovery teams, may receive, in real-time, correct coordinates for multiple moving people, all at once, assisting with precise and timely rescue.

It is also noted that the whole methodology is not limited in SAR applications, but seamlessly reflects other detection scenarios including environmental monitoring and pattern recognition (overgrazing, deforestation, etc.), vegetation identification for endangered species geo-location, animal observation, protection from poaching, etc.

When the moving platform speed is more than 20 km/h, a stabilizing system should be used to protect the UAV from flipping or crashing. Thus, for secure landing, electromagnets in landing legs or platform, suction systems or servo-actuated hooks are some solutions that currently are being tested by our group.

## Supplementary Materials

A “demonstration video” is available online at https://youtu.be/uj1OQxMQetA.
